# Direct observation of confined acoustic phonon polarization branches in free-standing semiconductor nanowires

**DOI:** 10.1038/ncomms13400

**Published:** 2016-11-10

**Authors:** Fariborz Kargar, Bishwajit Debnath, Joona-Pekko Kakko, Antti Säynätjoki, Harri Lipsanen, Denis L. Nika, Roger K. Lake, Alexander A. Balandin

**Affiliations:** 1 Phonon Optimized Engineered Materials (POEM) Center and Department of Electrical and Computer Engineering, University of California—Riverside, Riverside, California 92521, USA; 2 Laboratory for Terascale and Terahertz Electronics (LATTE), Department of Electrical and Computer Engineering, University of California—Riverside, Riverside, California 92521, USA; 3 Department of Micro and Nanosciences, School of Electrical Engineering, Aalto University, PO Box 13500, 00076 Aalto, Finland; 4 Institute of Photonics, University of Eastern Finland, FI-80101 Joensuu, Finland; 5 Department of Physics and Engineering, Moldova State University, Chisinau MD 2009, Republic of Moldova; 6 Spins and Heat in Nanoscale Electronic Systems (SHINES) Center, University of California—Riverside, Riverside, California 92521, USA

## Abstract

Similar to electron waves, the phonon states in semiconductors can undergo changes induced by external boundaries. However, despite strong scientific and practical importance, conclusive experimental evidence of confined acoustic phonon polarization branches in individual free-standing nanostructures is lacking. Here we report results of Brillouin—Mandelstam light scattering spectroscopy, which reveal multiple (up to ten) confined acoustic phonon polarization branches in GaAs nanowires with a diameter as large as 128 nm, at a length scale that exceeds the grey phonon mean-free path in this material by almost an order-of-magnitude. The dispersion modification and energy scaling with diameter in individual nanowires are in excellent agreement with theory. The phonon confinement effects result in a decrease in the phonon group velocity along the nanowire axis and changes in the phonon density of states. The obtained results can lead to more efficient nanoscale control of acoustic phonons, with benefits for nanoelectronic, thermoelectric and spintronic devices.

## Abstract (web summary)

In nanostructures, phonon confinement could lead to better control of phonon-electron coupling and thermal properties. Here, the authors use light scattering spectroscopy to measure acoustic phonons confinement in individual free-standing nanowires, their energy dispersion and energy scaling.

## Graphical abstract

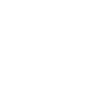

The phonon states in crystal lattices can undergo changes induced by external boundaries^1^,^2^. Modification of the acoustic phonon spectrum in structures with periodically modulated elastic constant or mass density—referred to as phononic crystals—has been proven experimentally and utilized in practical applications^3^,^4^,^5^,^6^,^7^,^8^. A possibility of modifying the acoustic phonon spectrum in individual nanostructures via spatial confinement would bring tremendous benefits for controlling phonon–electron interaction and thermal conduction at the nanoscale^9^,^10^,^11^. Engineering electron waves and energy dispersions with external boundaries in semiconductor heterostructures and quantum well superlattices became the foundation of modern electronics and optoelectronics^11^. Recent technological developments suggest that tuning the phonon energy dispersion may become as important for the next generation of nanoelectronic circuits as engineering of the electron dispersion. Acoustic phonons carry heat in semiconductors. Improving phonon transport in nanometer scale devices is crucial for their reliability. Phonons set performance limits for alternative technologies under development, from superconducting electronics to spintronics and quantum computing^12^,^13^.

It has long been suggested theoretically that sound waves in layered media^14^, or acoustic phonons in nanostructures, can undergo modification due to externally imposed periodic or stationary boundary conditions^15^,^16^,^17^. Modulation of the elastic constants, *C*_*ij*_, or mass density, ρ, in such structures can lead to changes in the acoustic phonon dispersion, *ω*(*q*), which include reduction in the phonon group velocity, *υ*_G_=∂*ω*(*q*)/∂*q* or emergence of the phonon energy band gaps (here *ω* and *q* are the phonon energy and wave vector, respectively)^5^,^8^,^10^,^15^,^16^,^17^,^18^,^19^. There are many experimental reports of the phonon wave effects in periodic structures, including the early Raman spectroscopy studies in semiconductor superlattices^20^. The possibility of controlling the acoustic phonon spectrum in periodic structures has led to an explosive growth in the field of phononic crystals^4^,^5^,^6^,^7^,^8^. Despite the strong motivations, and a large body of theoretical studies^10^,^11^, experimental investigation of the acoustic phonon confinement effects in individual nanostructures—as opposed to periodic composites—is lagging behind.

Establishing the existence of confined phonon subbands in free-standing nanostructures, and determining the length scale, *D*, at which they reveal themselves are important tasks. Controlling the phonon spectrum via spatial confinement would allow for fine-tuning of the phonon interactions with electrons, spins and other phonons, particularly at low temperature. A commonly used assumption in the thermal community^21^,^22^ is that the phonon spectrum modifications only appear when the structure size is on the order of the dominant thermal phonon wave length *λ*_T_≈(*υ*_s_*h*)/(*k*_B_*T*), which is about 1.5 nm for many solids at room temperature (RT) (here *k*_B_ is the Boltzmann constant, *T* is the absolute temperature, *h* is the Planck constant and *υ*_s_ is the sound velocity). This criterion is based on the notion that the phonon wave functions do not preserve coherence over larger distances owing to natural surface roughness in real nanostructures, defects and lattice anharmonicity. As a result, the phonon wave interference required for modification of the phonon dispersion does not take place even if solution of the elasticity equation predicts the appearance of confined phonon subbands. Another point of view^2^,^10^,^15^,^18^ considers that phonon spatial confinement effects start to take place when *D* is on the order of the average phonon mean free path (MFP), defined in kinetic theory as the grey MFP: *Λ*_G_=3*K*/(*C*_V_*υ*_s_), which is about 20 nm for bulk GaAs at RT (here, *K* and *C*_V_ are the thermal conductivity and specific heat capacity, respectively). This is considered of importance because energy differences between the phonon subbands in the middle of the Brillouin zone (BZ) can result in changes in the electron—phonon scattering, and, correspondingly, electron relaxation rates, particularly at low temperature^2^,^12^,^23^. In the thermal context, the spatial confinement and modification of the phonon density of states (DOS) can affect the thermal conductivity. Recent studies established that the phonon MFP can be substantially larger than *Λ*_G_ (refs 24, 25, 26). It was found, that 40% of thermal conductivity of crystalline Si near RT come from the phonons with MFP above 1 μm (ref. 26). The phonons with the longest MFP may have the lowest frequency, *ω*. The phonon life-time scales as *τ*∝*ω*^−s^, where *s*≥1 depends on the phonon scattering mechanism and dimensionality of the system. As a result, phonon MFP *Λ*=*υ*_G_*τ* will be growing with decreasing *ω*. The discovery of the large contribution of the long-MFP phonons to thermal conductivity makes the task of determining the length scale of the onset of confinement effects even more important.

Semiconductor NWs with smooth boundaries are the most suitable candidates for investigation of spatial confinement of acoustic phonons in the free-standing (or free-surface) nanostructures. For NWs, the characteristic length scale *D* is the diameter. However, random distribution of diameters in typical NW arrays, small distances between NWs, and difficulty of direct detection of acoustic phonons so far precluded confirming the theoretically predicted confined phonon branches in NWs^27^,^28^. A few published reports for NWs only infer possible confinement effects indirectly by measuring thermal conductivity and comparing the result with calculations, which use both bulk and confined phonon dispersion^29^,^30^. This approach is ambiguous owing to the difficulty of separating the phonon confinement effects from the phonon—boundary scattering. A Brillouin spectroscopy study of GaN nanowires made an important step forward in investigating confined phonons^31^. However, a large distribution in NW diameters, and discrepancy between the experimental and calculated dispersion left the question open. A Raman spectroscopy study of the etched Si membranes gave the energies of the slab phonon modes in the BZ center^32^. Another notable investigation of the etched suspended Si membranes confirmed the changes in the fundamental phonon modes but have not shown clear confined phonon subbands, possibly owing to surface roughness inherent for the etched films or strain in the suspended structures^33^.

Here we report measurements of the acoustic phonon spectrum of NWs in a unique set of NW arrays with different diameter and large inter-nanowire distance, which allowed us to conclusively prove the existence of the confined phonon polarization branches in individual nanostructures. We have also discovered that the phonon confinement effects become pronounced at a substantially larger length scale than previously believed. Our analysis is focused on confined phonons in the 4–40 GHz range. However, higher frequency confined phonons have also been observed. By varying the distance between the NWs we proved that the observed spectral feature are signatures of individual NWs. The measured phonon dispersion in NWs is in excellent agreement with theory. The obtained results can lead to more efficient nanoscale control of acoustic phonons, with benefits for various practical applications.

## Results

### Nanowire synthesis and characterization

**Figure 1:**
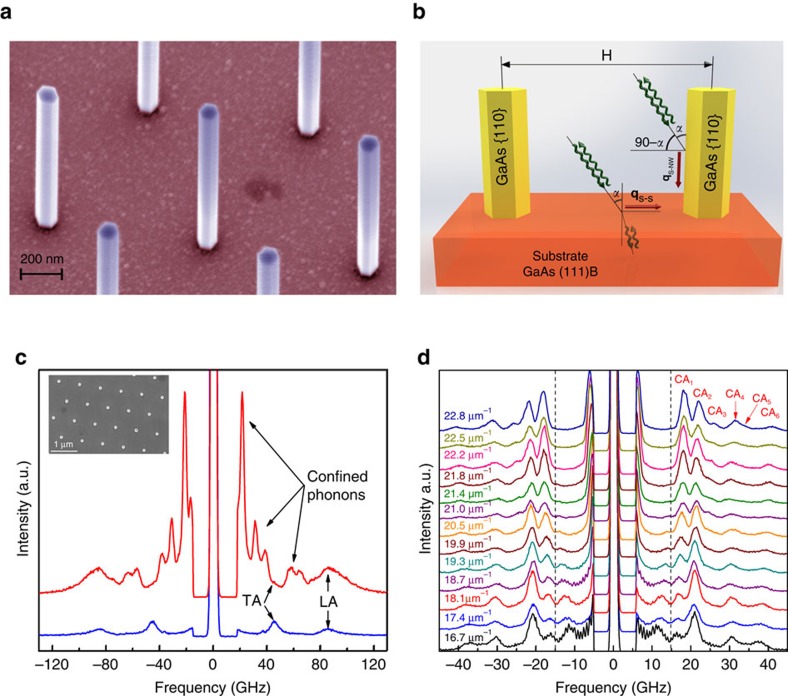
Free-standing GaAs nanowires and their acoustic phonon spectrum. (**a**) SEM image of NWs showing their diameter uniformity, orientation and large inter-nanowire distances (reaching up to *H*=3.0 μm in some samples). The pseudo-colours are used for clarity. (**b**) The schematic of the BMS experiment with notation for the substrate angle of incident *α*, which translates to *π*/2-α for NWs. Two mechanisms of light scattering—elasto-optic inside the substrate and ripple scattering from the side-facets of NWs and substrate surface—are illustrated with green arrows. (**c**) Measured phonon spectrum for NWs with the diameter *D*=122 nm (red curve) and a substrate without NWs (blue curve). The inset shows a top-view SEM image of a representative NW sample. The regular LA and transverse acoustic (TA) phonon peaks are present in both spectra. Additional peaks in the NW spectrum correspond to the confined acoustic phonons in individual NWs visible via the ripple scattering mechanism. These peaks appear at lower frequency than bulk phonons owing to the difference in the probing phonon wave vector *q*. (**d**) Evolution of the spectrum with the changing probing phonon wave vector defined by *α*. The confined phonon branches are denoted as CA_1_, CA_2_, and so on. Dash-lines are used to separate the peaks attributes to CA_*i*_ modes from the ones corresponding to NW's LA-like polarization branch for clarity. The spectral position of the peaks was accurately determined by the Lorentzian fitting.

Several sets of specially designed GaAs NWs with excellent surface quality and hexagonal cross section have been fabricated on top of a GaAs (111) substrate by selective-area epitaxy utilizing metal-organic vapour phase epitaxy (MOVPE). The top facets of the NWs have the same crystallographic plane as the substrate while the side-facets are {110} planes. The NWs were grown in a perfect vertical arrangement in hexagonal arrays with the diameters ranging from 103 nm up to 135 nm. The important attributes of the samples were diameter uniformity within each batch (the relative standard deviation was about 3% for most tested NWs), large distance, *H*, between the NWs (we focused on the range from 700 nm to 3 μm; the smallest *H* was 250 nm while the largest was 10 μm), and large length, *L*, of NWs (at least 10 times the NW diameter). A scanning electron microscopy (SEM) image of a representative sample is shown in Fig. 1a. Details of the sample fabrication and characterization are provided in the Methods, Supplementary Figs 1–3 and Supplementary Table 1.

### Brillouin-Mandelstam spectroscopy

We used Brillouin-Mandelstam light scattering (BMS) spectroscopy as a tool to measure the dispersion of acoustic phonons with energies in the range from 2 GHz up to 200 GHz near the BZ center. Various modifications of Brillouin spectroscopy^34^,^35^ are gaining popularity for investigating acoustic phonon and magnon energies in phononic crystals and other materials^31^,^36^,^37^,^38^,^39^,^40^,^41^,^42^,^43^,^44^,^45^. Changing the angle of light incidence, *α*, with respect to the substrate allowed us to vary the probing phonon wave-vector, *q*, and determine the dispersion near the BZ center. A schematic of the experiment is shown in Fig. 1b. The large inter-NW distance *H*, which was much larger than the laser wavelength, *λ*, ensured that NWs scatter light individually, for example, neither light interference nor elastic coupling via the substrate affect the results. We used the samples with *H* up to 3 μm to verify that the measured spectral characteristics are independent of *H*. Large *H* and *L*>>*D* also ensure that each NW can be modelled theoretically as an infinite NW with free-surface boundaries. These unique characteristics of NW samples made possible investigation of the phonon spectrum features originating in individual NWs.

There are three mechanisms, which contribute to light scattering in our samples. They are scattering from the bulk, that is, the volume of the substrate via the elasto-optic mechanism^34^,^35^; from the surface of the substrate via the surface ripple mechanism^34^,^35^; and from the side-facets of NWs via the surface ripple mechanism as well. In the volumetric mechanism (i) the phonon wave vector, *q*, is fixed at *q*_B_=4*πn*/*λ* where *n* is the refractive index of GaAs. In the ripple scattering mechanisms (ii) and (iii), the phonon wave vector is given by *q*_S−S_=(4*π*/*λ*)sin(*α*) for the substrate and *q*_S−NW_=(4*π*/*λ*)cos(*α*) for NWs (see notation for angle *α* in Fig. 1b). The difference in the angle dependence is due to the perpendicular alignment of NWs. In probing the ripple scattering mechanism, changing *α* allows one to vary *q* within a certain range. The volumetric elasto-optic scattering is absent in our NWs because *D<λ*. Although the light scattering is from the NW surface ripples it inherently depends on the confined phonon modes inside the NW, which create the ripples. In our experiments, changing α from 15° to 40° corresponded to 6.1 μm^−1^≤*q*_S−S_≤15.2 μm^−1^ and 18.1 μm^−1^≤*q*_S−NW_≤22.8 μm^−1^ in the ripple scattering from the substrate and NWs, respectively. Taking into account that *n*=4.13 for GaAs (ref. 46) at *λ*=532 nm the *q* value for the substrate elasto-optic scattering was fixed at *q*_B_=97.6 μm^−1^.

Figure 1c shows BMS spectra of NWs with the diameter *D*=122 nm (inter-NW distance *H*=800 nm) extracted from SEM data, and the substrate without NWs in the frequency range from 20 to 125 GHz. The phonon peaks at 45.6 and 85.8 GHz in the spectrum from the substrate correspond to the transverse acoustic and longitudinal acoustic (LA) polarization branches. These peaks originate from the true acoustic bulk phonons that have zero frequency at the BZ center, that is, *ω*(*q*=0)=0. The bulk phonon peaks are present in the spectra of NW samples as well because part of the signal is coming from the substrate. The most interesting feature of NW spectrum is the appearance of additional peaks attributed to the confined acoustic (CA) phonons. These phonons are quasi-optical in nature, in a sense, that their energy is non-zero at the BZ center, that is, *ω*(*q*=0)≠0. All confined phonons in NWs are hybrid in nature comprising of vibrations with different polarization. Although confined phonons are higher in energy than the true acoustic phonons, in the experimental spectrum, they appear at smaller frequencies because the probing phonon wave vectors are different: *q*_S−NW_<*q*_B_. Figure 1d presents the evolution of the BMS spectrum for the same NW sample for different values of *q* that are varied by changing the angle *α*. We were able to resolve six confined phonon branches denoted as CA_*i*_ (*i* is the confined phonon index). For clarity, the BMS spectra for the bare substrate without nanowires at various incident light angles has been shown in Supplementary Fig. 4. The peak below 15 GHz is a mixture of the true LA phonons in the substrate and LA-like phonons in NWs.

**Figure 2:**
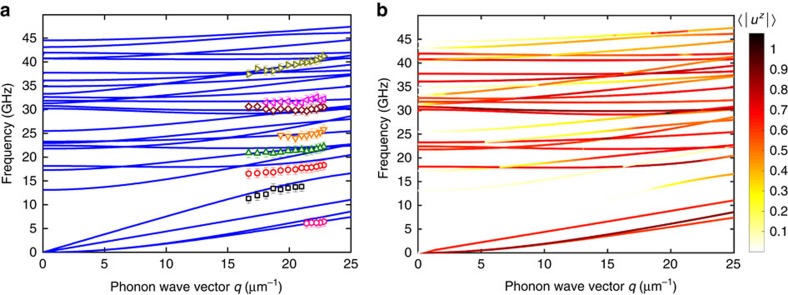
Confined acoustic phonon dispersion in semiconductor nanowires. (**a**) Measured and calculated phonon dispersion for a GaAs nanowire along [111] direction. The experimental data points, indicated with symbols and error bars, were obtained for NWs with the diameter *D*=122 nm determined from SEM inspection. Experimental uncertainties, on the order of 1 GHz, is within the standard deviation of the NW diameters (**b**) Calculated dispersion with the colour indicating the average surface displacement of the NW side-facet (〈|*u*^*z*^|〉) perpendicular to the phonon **q**_S-NW_. Darker colour corresponds to higher phonon mode activity in light scattering.

To understand and confirm the confined nature of the observed phonon peaks above 15 GHz, we solved the elasticity equation for NWs using the finite-element method. The simulations were carried out for NWs with hexagonal cross-sections using GaAs elastic constants for a specific crystallographic direction ([111]) and NW diameter determined from SEM. Figure 2a shows the simulated phonon dispersion (solid curves) in a hexagonal NW along the [111] direction for *D*=122 nm. One can see excellent agreement between the experimental data and modelling results for both true acoustic and confined phonon branches. The accurate BMS peak positions were determined using Lorentzian fitting (Supplementary Fig. 5). Experimental uncertainties, on the order of 1 GHz, is within the standard deviation of the NW diameters (that is, 2.9% for *D*=122 nm). Other sources of uncertainty are inevitable variations in the elastic behaviour of NWs resulting from the stacking faults and possible small inclusions of the wurtzite phase to the dominant zinc-blende (ZB) polytype in NWs with various diameters. This may result in a small change in the LA-like sound velocity *υ*_s_=(*C*_11_/*ρ*)^1/2^, defined by the slope of this branch. Additional analysis of the model sensitivity to parameter variations is provided in Supplementary Fig. 6.

## Discussion

Our proof of the confined nature of the phonons is based on excellent agreement of the dispersions obtained from BMS experiments and calculated for the exact NW shape and material parameters. In order to confirm that the identified phonon modes are Brillouin active, we calculated the average surface displacement of NW's side-facet perpendicular to *q*_S−NW_. In the surface scattering mechanism, the phonon modes that produce displacement perpendicular to *q*_S−NW_ in the plane of scattering are those likely to contribute to the BMS spectrum. The scattering cross-section is given by (ref. 47)


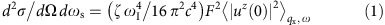


Here ζ is a coefficient proportional to the illuminated area, *ω*_I_ is the frequency of the incident light, *c* is the speed of light in the vacuum, *F* is a function of the incident and scattered light angles as well as *n* of the scattering medium. The last term <…> is the surface-displacement power spectrum with *q*_x_ projection to the surface. The calculated displacements, which enter equation (1), are shown in Fig. 2b by the colour of the simulated phonon branches. The results are in agreement with the experiment in a sense that the measured data points are all within the Brillouin active segments of the polarization branches. The fact that these branches are active is in line with the observation that the considered phonon modes are hybrid in nature.

**Figure 3:**
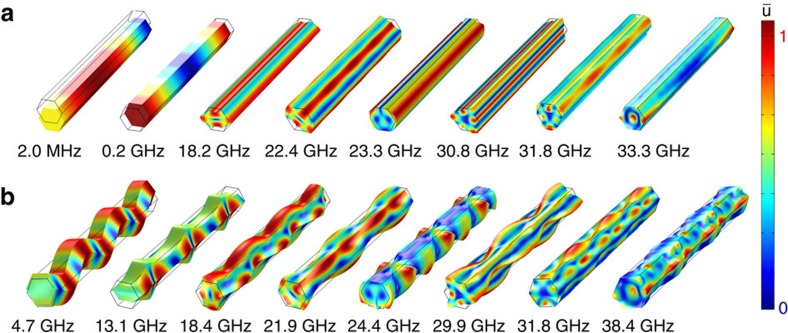
Normalized displacement field of the Brillouin-active phonon modes. The calculated data are for a 1-μm long NW with (**a**) *q*_S-NW_=0.3 μm^−1^ and (**b**) *q*_S-NW_=18.0 μm^−1^. The red colour corresponds to stronger displacement.

The phonon modes are visualized in Fig. 3a,b as the normalized displacement field distributions in NWs for two wave-vectors *q*_S−NW_=0.3 μm^−1^ and *q*_S−NW_=18.0 μm^−1^—close and away from the BZ center, correspondingly. The modes become more hybrid in nature as *q* increases. The active modes show strong surface ripple on the side facets of NWs. The symmetry of the confined modes is complicated. The point group of ZB is *T*_d_ with 24 invariant operations whereas wurtzite is characterized by *C*_6v_ (ref. 48). Confined NW geometry reduces the symmetry to *C*_3v_. The two lowest branches are flexural acoustic modes of *E*_1_ symmetry with the quadratic dispersion *ω*∝*k*^2^. The degeneracy of this branch is broken for NWs along the [111] direction. The next two acoustic modes, torsional acoustic of *A*_2_ symmetry and LA-like of *A*_1_ symmetry, respectively, have linear dispersion *ω*∝*k*, near the BZ center. The confined branches mainly belong to the higher symmetry groups for example, *E*_2_, *B*_1_, *B*_2_. See animation of the modes in Supplementary Movie 1.

**Figure 4:**
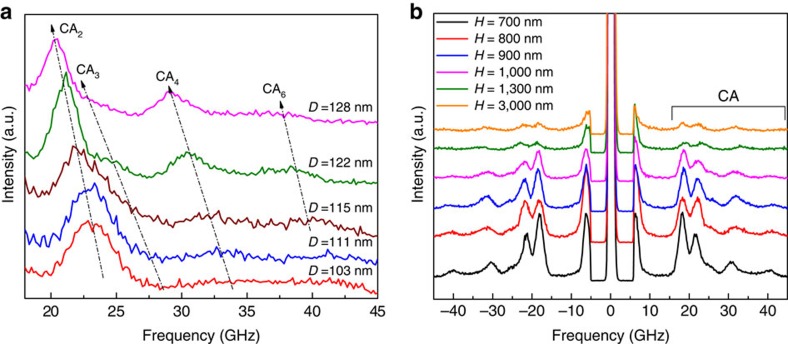
Effect of the diameter and inter-nanowire distance on confined phonon energies in nanowires. (**a**) Brillouin-Mandelstam spectrum for NWs with different diameter at a constant probing phonon wave vector *q*_S-NW_=18.1 μm^−1^. The decrease in the frequency of the confined phonons with increasing NW diameter is clearly visible. The confined phonon branches show strong diameter dependence even for relatively large *D* values in the range from 103 to 128 nm. The diameter dependence proves conclusively the presence of the spatial confinement effects at the length scale above the grey phonon mean-free path. (**b**) Measured spectrum for a set of NWs with the constant diameter *D=*122 nm and varying inter-NW distance *H* at a constant probing phonon wave vector *q*_S-NW_=22.8 μm^−1^. The data are presented for the same fixed accumulation time of 30 min. The spectral position of the CA peaks does not depend on *H*. The intensity decreases with increasing *H* owing to smaller number of illuminated NWs. The absence of the inter-NW distance dependence proves that the observed spectral features are characteristics of individual NWs.

The final element of evidence of spatial confinement of acoustic phonons in free-standing NWs comes from the analysis of the diameter dependence of the CA phonon frequencies (energies). Figure 4a shows the measured phonon spectra for different NW diameters, *D* (*H*=800 nm for all samples). One can see that with increasing *D*, the frequencies of the confined phonons decrease. The trend and the magnitude of the frequency change are consistent with the theory (see calculated dispersions for various *D* in the Supplementary Fig. 7). In Fig. 4b we present spectra of NWs with the constant *D*=122 nm and varying *H*. The spectral position of CA peaks does not depend on the inter-NW distance. The absence of *H* dependence indicates that the measured spectral features are characteristics of individual NWs. There is no elastic coupling among NWs. The spatial phonon confinement in the free-surface NWs is distinctively different from the phonon spectrum changes owing to the periodic boundary conditions in the phonon band-gap materials.

A remaining intriguing question in confirming the confined nature of CA peaks is why their full-width-at-half-maximum (FWHM) appear smaller than that of the substrate LA phonon peak (see Fig. 1c). Theory suggests that the phonons with smaller group velocity, *υ*_G_, should be more strongly scattered^18^. The phonon life-time limited by the point-defect scattering, 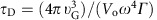, rapidly decreases with decreasing *υ*_G_ (here *V*_o_ is the volume per atom and *Γ* is the defect scattering factor). The Umklapp limited phonon life-time also decreases with decreasing *υ*_G_. The answer to this question is that FWHM of the elasto-optic and ripple mechanism peaks cannot be compared directly. The FWHM of the LA peak from the substrate (*Δω*=13.6 GHz) is defined by the light absorption. For an opaque crystal with the refractive index *n*=*n*_1_+*in*_2_, theoretical broadening for the elasto-optic scattering^49^ is *Δω*/*ω*=2*n*_2_/*n*_1_. Using for GaAs, *n*_1_=4.13 and *n*_2_=0.34 [46], we obtain *Δω*/*ω*=0.164, which closely matches with the measured *Δω*/*ω*=0.158. For the CA phonons, observed in our experiments via the surface ripple scattering, the peak broadening is defined by the aperture effects^34^,^35^.

The fact that the acoustic phonon spectrum becomes strongly modified near BZ center at the length-scale much larger than the grey phonon MFP or the thermal phonon wavelength has important implications. It means that the acoustic phonon confinement can affect the electron—phonon scattering rates^50^ in the structures comparable in size to the state-of-the-art electronic devices. The BZ-center phonons (*q*<10^6^ cm^−1^) are essential for controlling electron relaxation^2^,^15^,^17^,^23^. The frequency-dependent average phonon group velocity^51^ 〈*υ*(*ω*)〉=*g*(*ω*)/Σ_*j*_1/*υ*_*j*_(*ω*), which affects the heat conduction, also changes (here *g*(*ω*) is the number of phonon branches in the spectra with the frequency *ω*, and *υ*_*j*_(*ω*) is the group velocity of the phonon mode of the subband *j* and frequency *ω*. The group velocity of each phonon branches is shown in Supplementary Fig. 8a). In NWs with *D*∼100 nm, 〈*υ*(*ω*)〉drops to less than half of the LA-like mode sound velocity in the frequency range around 20–60 GHz due to emergence of the confined phonon branches.

Our measurements were conducted in the GHz range of phonon frequencies, which dominates heat conduction at low temperature^52^. The fact that we established the existence of the confined phonon branches at RT indicates that the effects can be even stronger at low temperature. THz phonons are considered to be the main contributors to thermal transport at RT owing to a much higher DOS, which scales as *ω*^2^ in bulk crystals. However, the nearly dispersion-less confined phonons in the BZ-center can substantially change the DOS (Supplementary Fig. 8b) resulting in a greater role for the GHz phonons in thermal transport. Even in bulk, the low-frequency phonons, including the GHz range, participate in the phonon—phonon scattering processes^1^,^53^. The role of the low-frequency phonons is greatly increased in alloyed materials because the higher frequency phonons scatter more strongly^54^. A possibility of reducing the phonon thermal conductivity in nanostructures with smooth boundaries is important for thermoelectric devices. The phonon confinement mechanism allows one to reduce the thermal conductivity without degrading the electron transport. This may become particularly relevant in the quest for efficient low-temperature thermoelectric materials.

## Methods

### Sample preparation

GaAs NWs were fabricated with selective-area epitaxy using MOVPE system (Thomas Swan)^55^. The growth was performed on p-type GaAs (111) B substrates in atmospheric pressure. Before NW growth the growth templates were fabricated as follows. First, a 40-nm thick SiO_*x*_ layer was deposited using the plasma-enhanced chemical vapour deposition (Oxford Systems). Next, electron beam lithography (Vistec) was performed to pattern the growth templates and reactive-ion etching was used to transfer the patterns to the deposited SiO_*x*_ layer. Poly(methyl methacrylate) (MicroChem) was used as the electron beam lithography resist. The patterns contained triangular lattice arrays of circles with different diameters and pitches. Each array of circles was 100 μm by 100 μm in size and the diameter of the circles was varied from 40 nm up to 125 nm with a 5 nm step. The pitch was varied from 250 nm up to 10 μm with minimum step size of 50 nm. Before transferring the samples into the MOVPE reactor, resist stripping, degreasing and cleaning were performed in acetone, isopropanol and de-ionized water. The NW growth was performed using trimethylgallium (TMGa) and tertiary-butyl arsine (TBAs) as the precursors with H_2_ carrier gas and total gas flow of 5 slm. The molar flows were 0.811 μmol min^−1^ and 226.1 μmol min^−1^ for TMGa and TBAs, respectively. The samples were thermally cleaned at 760 °C under TBAs flow for 5 min just before initiating NW growth by turning TMGa flow on. The growth temperature was the same as for the thermal cleaning. Three samples were fabricated with different NW growth time. The growth times were 6 min, 11 min 30 s and 15 min. This was done to acquire approximately equal height for smaller and larger diameter NWs. When the growth was finished, the TMGa flow was cutoff and the samples were cooled to 150 °C under TBAs protection. SEM images of the samples with different diameters and pitches are shown in Supplementary Figs 1,2 and 3.

### Brillouin-Mandelstam spectroscopy

The experiments were carried out in backscattering geometry with p-polarized incident light using a solid-state diode pumped laser operating at *λ*=532 nm. The laser light was focused on the samples through a lens with NA=1.4. The scattered light was collected with the same lens and directed to the high-resolution six-pass tandem Fabry-Perot interferometer (JRS Instruments). A specially designed stage allowed to rotate the samples up to 60° relative to the direction of the incident laser light with an accuracy of 0.02°.

### Finite-element method simulations

The phonon dispersion and displacement patterns have been calculated in the elastic continuum approximation using FEM implemented in COMSOL Multiphysics package. The GaAs sample is assumed to have ZB crystal structure. The out-of-plane direction of the nanowire is along *x*, which is also the growth direction (that is, [111]). From the second-order continuum elastic theory, the equation of motion for the elastic vibration is


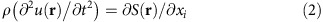


Here u(**r**) is the three-component displacement vector at coordinate **r**; *ρ* is the mass density; *S*(**r**) is stress tensor that can be constructed from displacement by *S*_*ij*_*=C*_*ijkl*_ *ɛ*_*kl*_ with the six-component elastic strain tensor^56^, ɛ(**r**)=½[(∇**u**)^T^+(∇**u**)]. The fourth-ranked elastic stiffness tensor, *C*_*ijkl*_, is also expressed in the non-tensor notation as *C*_*ij*_ with indices *i, j, k, l* running over the spatial coordinates (*x, y, z*). The elastic constants, *C*_*ij*_, used in simulation correspond to GaAs in [001] direction: *C*_11_=118.8 GPa, *C*_12_=53.8 GPa, *C*_44_=59.4 GPa (ref. 48). According to *ab initio* simulations^48^ and experiment^48^, the bulk stiffness tensor is equally applicable to nanowire geometry as long as the nanowire diameter is not smaller than 20 nm. In order to take into account the growth direction of the nanowire we applied the tensor rotation operation to transform the stiffness matrix in [111]-oriented coordinate system as


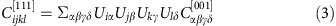


Here *U* is the rotational matrix for the Euler angles. The transformed elastic matrix and material parameters are summarized in Table 1. The simulation geometry is discretized using finite element scheme to obtain the solution of the elasticity equation in the frequency domain as- *ρω*^2^*u*=∇·***S***, where *ω* is the Eigen frequency. The free surface boundary conditions are applied at all outer facets in the radial direction by setting *ɛ*_*ij*_*n*_*j*_=0, where *n*_*j*_ is the outward normal unit-vector.

### Data availability

The data that support the findings of this study are available from the corresponding author upon request.

## Additional information

**How to cite this article:** Kargar, F. *et al*. Direct observation of confined acoustic phonon polarization branches in free-standing semiconductor nanowires. *Nat. Commun.* **7**, 13400 doi: 10.1038/ncomms13400 (2016).

**Publisher's note:** Springer Nature remains neutral with regard to jurisdictional claims in published maps and institutional affiliations.

## Supplementary Material

Supplementary Information Supplementary Figures 1-8, Supplementary Table 1 and Supplementary Methods.

Supplementary Movie 1 Normalized displacement field of the Brillouin-active phonon modes. The animation shows the displacement field of confined acoustic phonon branches for a 1-μm long NW at q_S-NW_ = 0.3 μm^-1^ (close to BZ center) and q_S-NW_ = 18.0 μm^-1^ (away from BZ center). The red color corresponds to stronger displacement.

## Figures and Tables

**Table 1:** Material parameters.

***C***_**11**_ **(GPa)**	***C***_**12**_ **(GPa)**	***C***_**13**_ **(GPa)**	***C***_**15**_***=−C***_**25**_***=−C***_**46**_ **(GPa)**	***C***_**33**_ **(GPa)**	***C***_**44**_ **(GPa)**	***C***_**66**_ **(GPa)**	***ρ*** **(kg** **m**^**−3**^**)**	***ɛ***_**r**_
145.7	44.8	35.8	12.7	154.6	41.5	50.4	5,317	13.18
