# Supplementary material for: Direct observation of confined acoustic phonon polarization branches in free-standing semiconductor nanowires
Source: Nat Commun. 2016 Nov 10;7:13400. doi: 10.1038/ncomms13400 (PMC5109588; doi:10.1038/ncomms13400)
Supplement: Supplementary Information — Supplementary Figures 1-8, Supplementary Table 1 and Supplementary Methods. [file ncomms13400-s1.pdf]

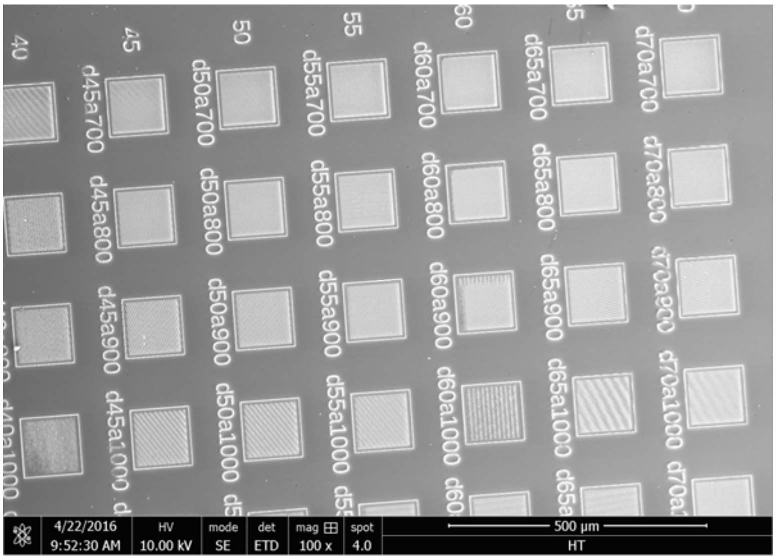

Supplementary Figure 1 | SEM image of the sample with different NW diameters and inter-nanowire distances. The lithographic diameter of the nanowires and inter-nanowire distances are denoted as *d* and *a*, respectively.

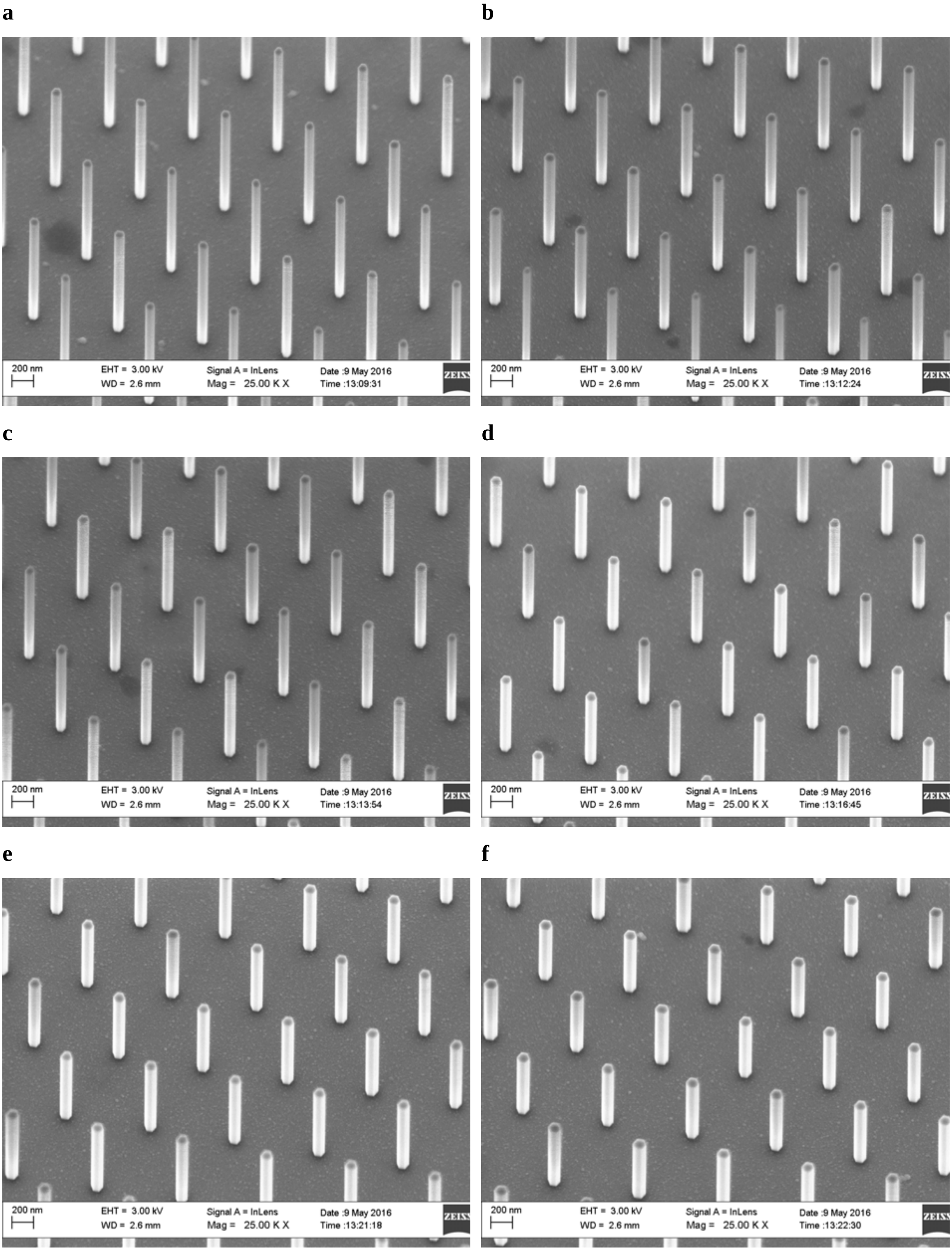

Supplementary Figure 2 | Side-view SEM images of samples. Detailed SEM analysis has been performed on all samples in order to determine the actual NW diameter, *D*, Length, *L* and distance, *H*, between the NWs. The representative data are for samples with the same *H*=800 nm and different diameter of (a) *D*=103 nm, (b) *D*=111 nm, (c) *D*=115 nm, (d) *D*=122 nm, (e) *D*=128 nm, and (f)

D=135 nm. The ImageJ software was used to determine the length, *L*, and diameter of individual NWs. For each sample more than 20 measurements were performed. The NW size data, including the standard deviation, are summarized in Supplementary Table 1.The results confirm the excellent quality and uniformity of the samples. Each batch contained NWs with almost the same diameter and length without significant variations.

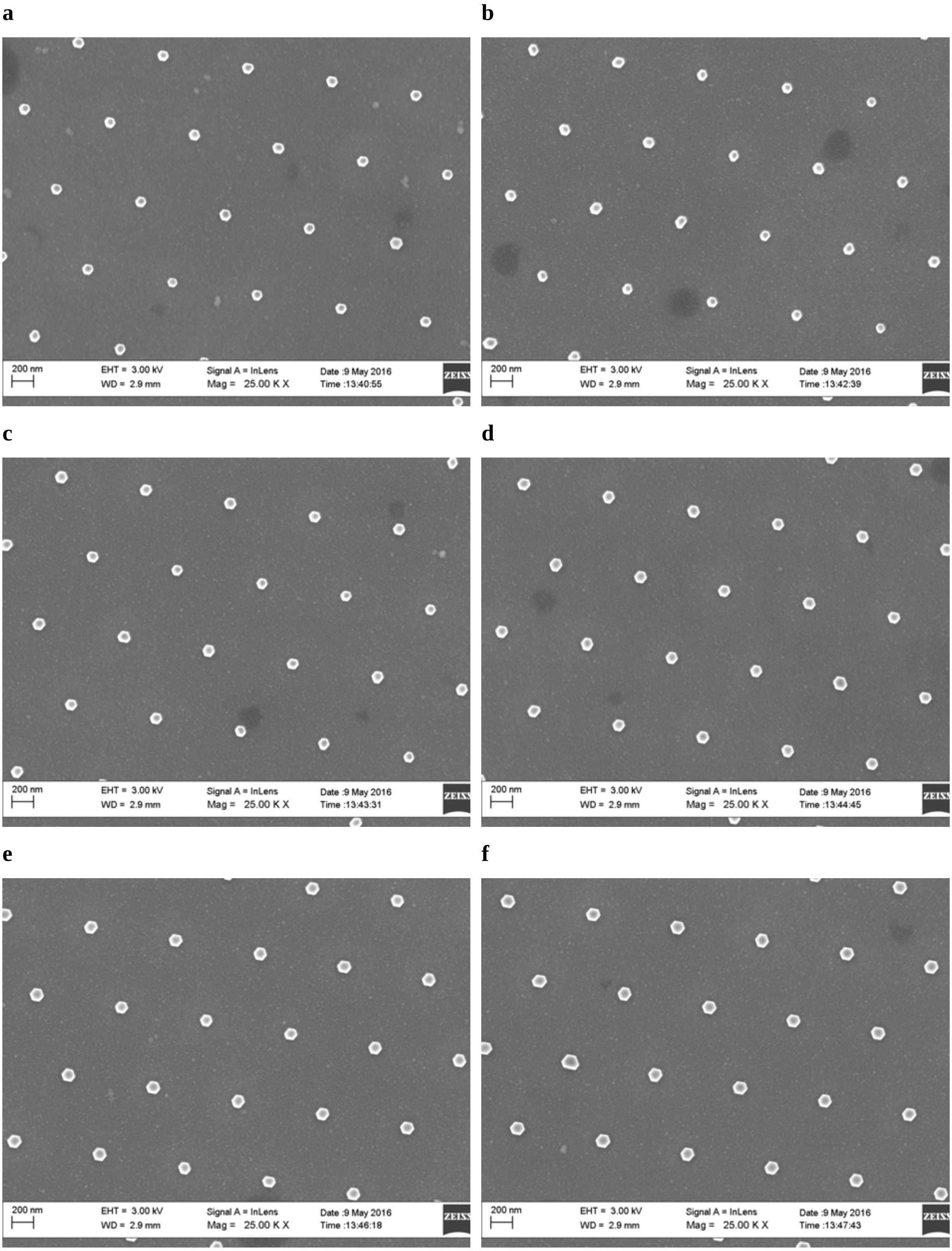

Supplementary Figure 3 | Top view SEM images of samples. (a) D=103 nm, (b) D=111 nm, (c) D=115 nm, (d) D=122 nm, (e) D=128 nm, and (f) D=135 nm.

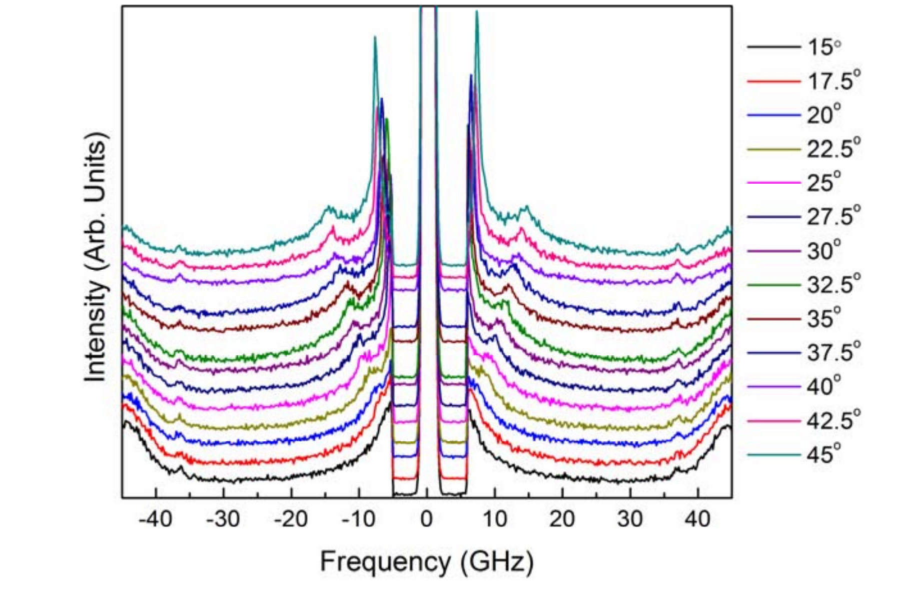

Supplementary Figure 4 | BMS spectrum of the reference substrate. In order to be able to distinguish and exclude, if needed, the effect of the substrate, BMS has been conducted at different incident angles on a reference substrate without NWs. As it can be seen, as the incident angle increases, the small peak which originates from the surface ripple scattering of the substrates surface moves to higher energies. At incident angles of ~40° or higher, this peak mixes with the true LA mode of the NWs and makes the spectrum noisy. At incident angles close to this value, another sharp peak appears at ~5 GHz which corresponds to GaAs surface Rayleigh wave. The angles for the study of NWs were selected to minimize the substrate effects and facilitate the data analysis.

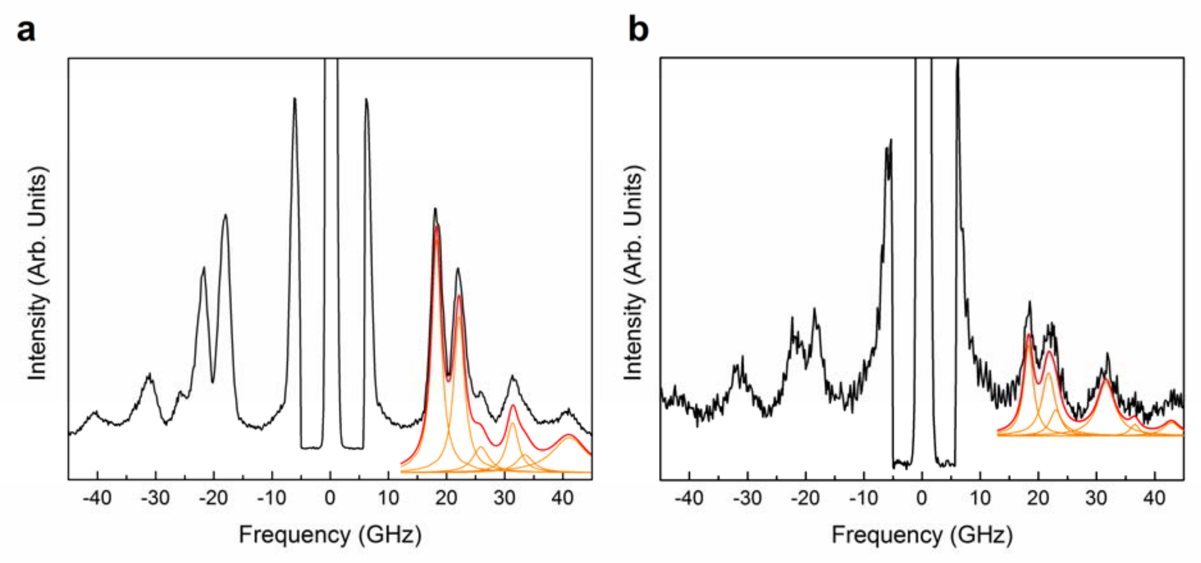

Supplementary Figure 5 | Examples of BMS spectrum analysis. An example of deconvolution for NWs with diameter D=122 nm and inter-nanowire distance (a) H=800 nm and (b) H=3000 nm is presented for constant phonon wave-vector of $q_{S\text{-}NW}$=22.8 μm^{-1}. In most of cases, the measured confined phonon and true acoustic phonon peaks were clearly observed. They could be fitted with individual Lorentzian functions. However, some peaks were two close to each other to be visually resolved. In these cases, peak deconvolution with several Lorentzian functions was used. The positions of the Lorentzian functions were then checked using the calculated phonon dispersion. As one can see, increasing the inter-NW distance does not change the position of the peaks. The signal-to-noise ratio decreases (fewer NWs are illuminated) making fitting less accurate. The date proves that NWs scatter light individually. For both spectra, while the first two peaks are very sharp the peak at ~35 GHz is broad. For the broad peak, a standard deconvolution procedure has been used. One can see that the broad peak consists of two elemental peaks close to each other. The orange lines in this figure are single Lorentzian functions while the red line represents the cumulative fitting. The positions of the elemental Lorentzian functions used in the decomposition are in excellent agreement with the calculated dispersion.

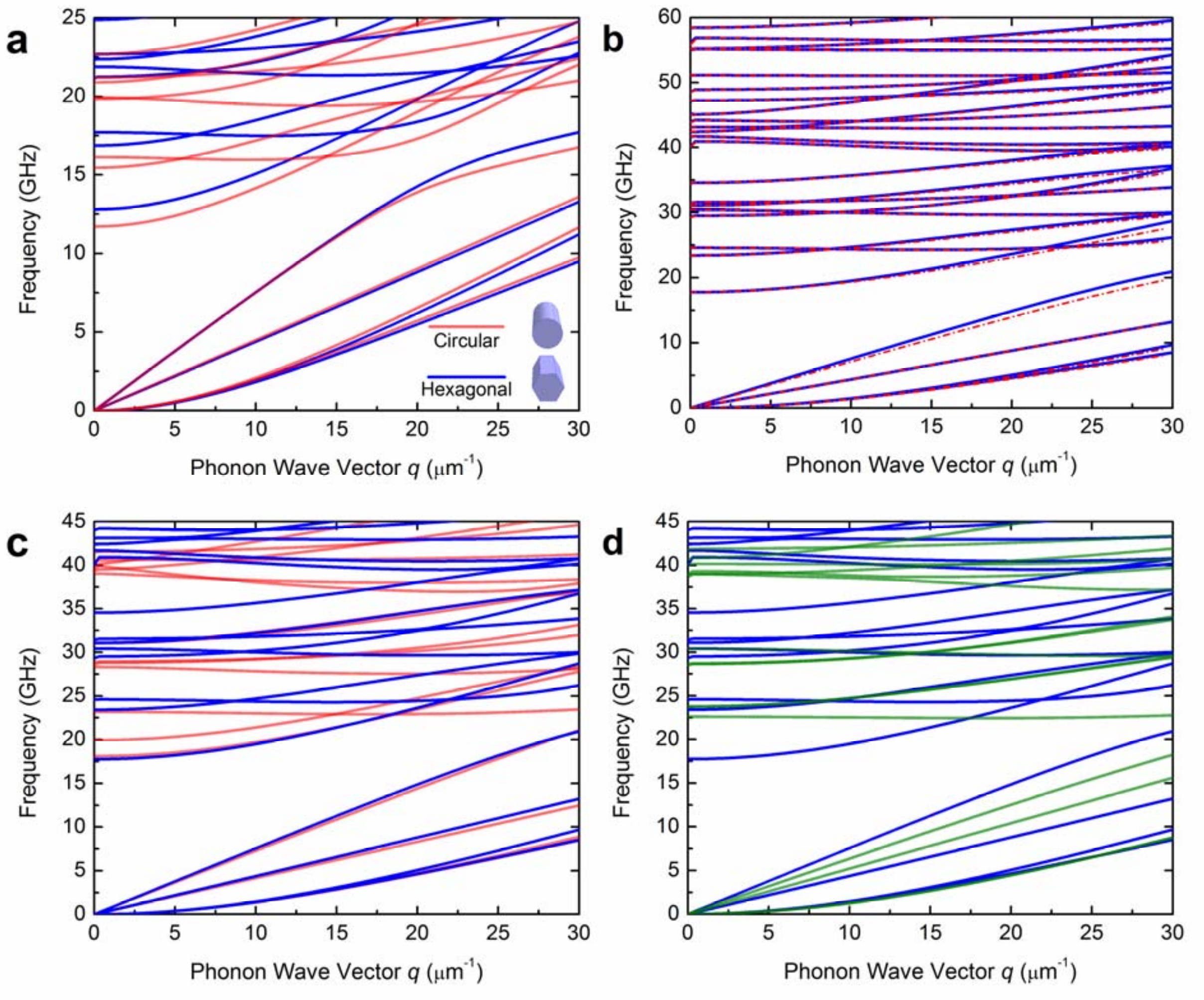

Supplementary Figure 6 | Effect of different parameters on calculated phonon dispersion of NWs. (a) Sensitivity of the phonon dispersion to the NW cross-section shape. The red and blue curves correspond to the circular and hexagonal cross-section, correspondingly. The data were calculated for NW diameter D=125 nm and crystal orientation along [111]. One can see that depending on the NW cross-section shape the confined phonon modes can deviate by ~1.5 GHz to 2 GHz, which can be resolved experimentally. (b) Effect of stiffness coefficient (matrix element C_{11}) on phonon dispersion. The longitudinal acoustic (LA) sound velocity may vary slightly from sample to sample due to growth condition and residual strain. This can be reflected in numerical modeling via changes in the C_{11} value. One can see that tuning C_{11} by 10% affects slightly the LA-like mode but does not change the BMS active confined acoustic (CA) mode. This is in line with our experimental observation that the BMS peaks of confined mode frequencies remain same for different sample with fixed D and cross-section. (c) Sensitivity of the phonon dispersion to crystalline structure by simulating it for zinc-blende (ZB) (blue curve) and wurtzite (WZ) (red curve) phases of NW with D=90 nm. (d) Influence of the crystal orientation on the phonon dispersion in ZB GaAs NW with D=90 nm. Changing orientation reduces the original point group symmetry. This results in the breaking of degeneracy in FA modes (lowest two branches). The group velocity of LA-like branch also depends strongly on crystal orientation. This explains why the data on crystal orientation of NW samples were important for interpretation of the experimental data. The blue and green curves represents the data for zinc-blende [111] and zinc-blende [001] directions, respectively.

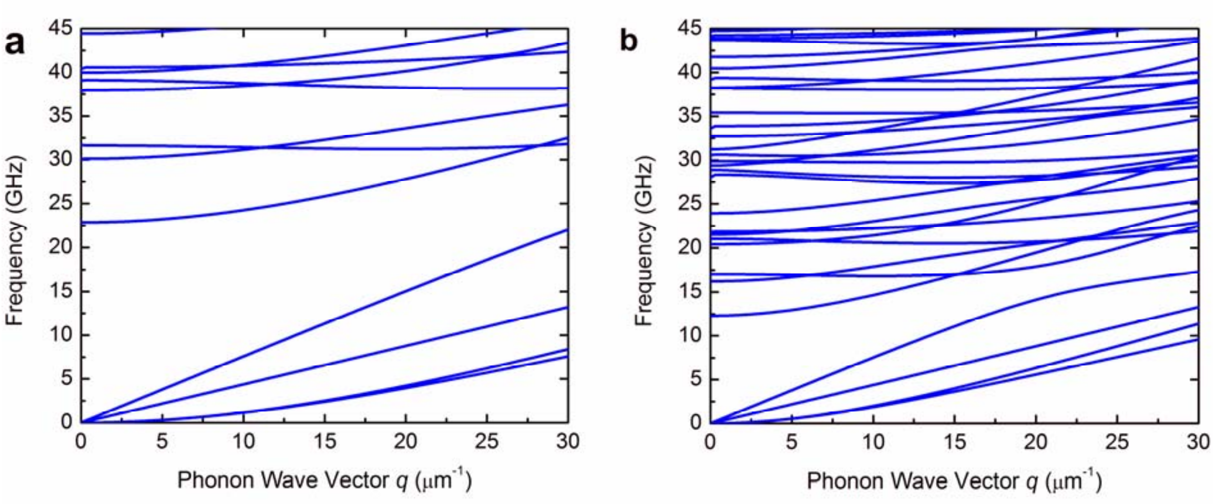

Supplementary Figure 7 | Calculated phonon dispersion for NWs with different diameters. A comparison of phonon dispersion of GaAs NW along [111] direction for different diameter (a) D = 70 nm and (b) D = 130 nm is presented. At small values of q<15 μm^{-1}, the fourth phonon branch is LA-like and demonstrates sufficiently high group velocity. The increase of q leads to the confinement induce changes in polarization (the phonon modes become mixture of LA and TA vibrations) with the corresponding decrease in its group velocity.

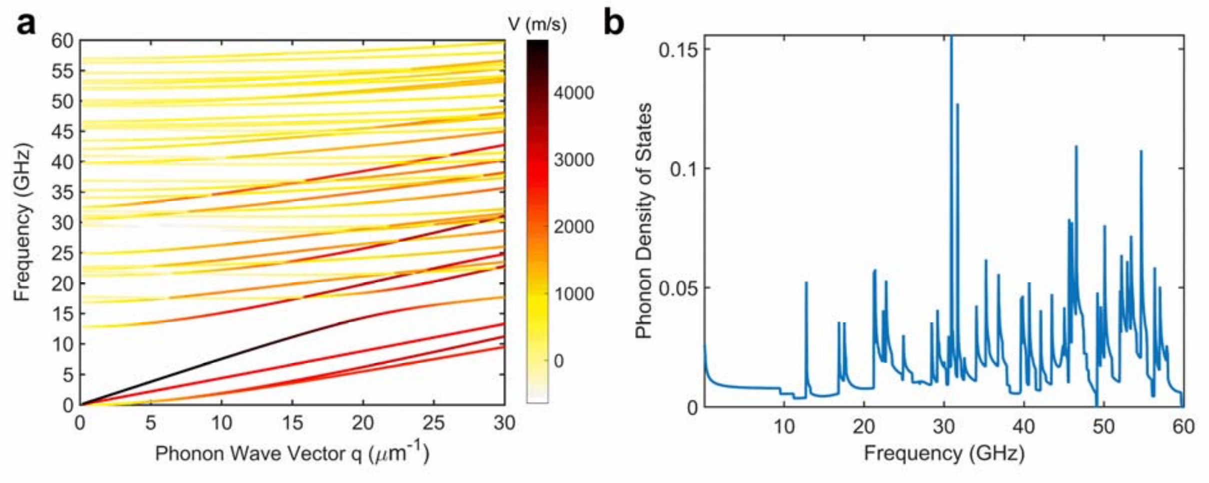

Supplementary Figure 8 | Calculated phonon group velocity and density of states. (a) Calculated phonon dispersion indicating the relative contribution of each branch to the mode-averaged phonon group velocity. The change in the color from yellow to black corresponds to increasing contribution to the mode-average phonon group velocity. (b) Calculated phonon density of states. The simulations were performed for D = 125 nm. The data indicates that the effect of the confined phonons is significant, leading to reduction of the average group velocity. The phonon density of states is modified as compared to bulk owing to the emergence of the confined phonon bands.

Supplementary Table 1. Measurement of diameter and length of NWs based on SEM images

Diameter *D* (nm)	Relative Standard Deviation (%)	Length *L* (nm)	Relative Standard Deviation (%)
103	4.2	2058.6	4.8
111	5.7	1977.1	5.6
115	4.6	1716.5	4.4
122	2.9	1448.9	4.1
128	3.7	1277.3	3.3
135	1.7	1127.5	3.0

Supplementary Methods

Brillouin-Mandelstam Spectroscopy. The experiments were carried out in backscattering geometry with p-polarized incident light using a solid-state diode pumped laser operating at λ=532 nm. The laser light was focused on the samples through a lens with diameter of 25.4 mm and focal distance of 35.0 mm (*NA*=1.4). The laser power on the sample were maintained the same for all experiments as low as possible (23 mW) in order to avoid self-heating effects. The scattered light was collected with the same focusing lens and directed to the high-resolution six-pass tandem Fabry-Perot interferometer (JRS Instruments). A specially designed stage allowed us to rotate the samples up to 60° relative to the direction of the incident laser light with an accuracy of 0.02^{o}. In order to get as many data points as possible, the angle of incident adjusted to 15° ($q_{S\text{-}NW}$ = 22.8 µm^{-1}) at the beginning and then increased by 2.5^{o} steps for changing the probing phonon wave-vector. Depending on a specific sample, 11 to 15 different data points have been stored.
